# Kinetics of non-structural protein 1, IgM and IgG antibodies in dengue type 1 primary infection

**DOI:** 10.1186/1743-422X-8-47

**Published:** 2011-02-02

**Authors:** Dongmei Hu, Biao Di, Xixia Ding, Yadi Wang, Yue Chen, Yuxian Pan, Kun Wen, Ming Wang, Xiaoyan Che

**Affiliations:** 1 Center for Clinical Laboratory, Zhujiang Hospital, Southern Medical University, Guangzhou, PR China; 2 Center for Disease Control and Prevention of Guangzhou, Guangzhou, PR China

## Abstract

**Background:**

Early and accurate diagnosis of dengue infection is essential for control of disease outbreaks. Recently, the dengue virus non-structural antigen 1 (NS1), a conserved and secreted glycoprotein, has been used as a marker for early diagnosis of dengue with convenience and cost-effectiveness. Serological tests of dengue IgM and IgG antibodies are still the most widely used for diagnosis of dengue. In order to assess combined diagnostic value of these tests, we study the kinetic profiles of circulating NS1, dengue IgM and IgG antibodies over the course of the disease by using an in-house dengue type 1 (DENV1) specific NS1 capture ELISA and the commercial Panbio Dengue IgM and IgG capture ELISAs.

**Results:**

A panel of 313 acute-and early convalescent-phase serum specimens from 140 DENV1 primary infected patients during an outbreak of dengue in Guangzhou, China, in 2006 were studied. Dengue NS1 presented high levels in acute-phase serum samples. It was detectable as early as day 1 of illness, and up to 14 day after onset. The sensitivity of NS1 detection was ranged from 81.8% to 91.1% with samples taken during the first 7 days. Anti-dengue IgM antibody was detectable on the third day of onset with the positive rate of 42.9%, and rapidly increasing to 100% by day 8 of illness. Anti-dengue IgG antibody was detectable on the fifth day of onset with low level at the first week of onset, and slowly increasing to 100% by day 15 of illness. Combining the results of NS1 and IgM antibody detection allowed positive diagnosis in 96.9% -100% for samples taken after day 3 of onset.

**Conclusions:**

Dengue NS1 detection might shorten the window period by first few days of illness. A combination of dengue NS1 antigen and IgM antibody testing facilitates enhanced diagnosis rates. The procedures should be suitable for developing countries where dengue is endemic.

## Background

Dengue is a major public health concern globally [1]. The incidence rate of the disease increased rapidly during the last decades. Dengue virus (DENV) consists of four distinct serotypes (DENV1 to 4). Infection with any one of the serotypes can cause a broad spectrum of manifestations from asymptomatic or mild dengue fever (DF) to dengue hemorrhagic fever (DHF) or dengue shock syndrome (DSS). As no protective vaccine or specific treatments are available for dengue, early and accurate laboratory diagnosis is essential for the effective surveillance and control of disease outbreaks. Currently, dengue diagnostic methods are based on virus isolation, RNA and antigen detection, and serology [2,3]. Viral RNA detection assays provide a highly sensitive and rapid diagnosis in the acute phase, but this approach requires specialized laboratory equipments and experienced technicians which are limitations in many developing countries where dengue is endemic [4]. IgM antibody capture enzyme-linked immunosorbent assay (MAC-ELISA) is the most commonly used technique for routine diagnosis. The dengue serological assays however become more challenging because dengue antibodies are cross reactive with other flaviviruses such as West Nile virus (WNV), St. Louis encephalitis virus (SLE), Japanese encephalitis virus (JEV), and yellow fever virus (YFV). In addition, IgM antibody response varies considerably among the individuals due to host humoral immune response or depending on whether a primary *vs *a secondary infection [2,4]. More recently, dengue virus non-structural protein 1 (NS1) antigen capture ELISAs have been reported as being a promising tool for the diagnosis of acute dengue infections [5-12]. NS1 antigen assay has many advantages over RT-PCR assays including rapidity, convenience and cost-effectiveness. Circulating NS1 has been shown to be detectable from the first day to the early convalescent phase after onset of disease. Monoclonal antibody (MAb)-based serotype-specific NS1 assays can be used to differentiate between flaviviruses [8,10].

ELISA-based detection of viral antigens and specific antibodies have the advantage of being easier to perform and standardize, specially being suitable for resource poor countries. Consequently, these procedures are likely to become routine methods for diagnosing dengue infection. An understanding of the kinetic profiles of dengue NS1, as well as dengue IgM and IgG antibody responses will help clarify the advantages and disadvantages of these tests for diagnosing dengue infection. In this study, we used a well-characterized panel of acute and early convalescent-phase serum specimens collected from dengue patients during DENV1 outbreak in Guangzhou, China, in 2006 to study the kinetic profiles of circulating NS1, dengue IgM, and IgG antibody responses over the course of the disease. The aim of the present study was to evaluate combined diagnostic value of these tests.

## Materials and methods

### Clinical samples

A panel of 313 acute- and convalescent-phase serum specimens were collected between days 1 and 27 after the onset of symptoms from 140 infected patients during the disease outbreak in Guangzhou, Guangdong province, China, in 2006 [13,14]. All these patients had been laboratory-confirmed previously as being infected with DENV1 by virus isolation and/or viral RNA detection by RT-PCR and/or serological diagnosis by MAC-ELISA. Of these 140 patients, 109 patients provided two serum samples; 29 patients had three serum samples, and 2 patients had four serum samples. All the patients were classified as having dengue fever; no patient had the severe manifestations of dengue hemorrhagic fever or dengue shock syndrome, according to the World Health Organization criteria [15]. Disease day 1 was designated as the day of the onset of symptoms. Five hundred and thirty-seven normal serum specimens from healthy donors were used as negative controls.

### NS1 detection with DENV1 specific NS1 capture ELISA

Detection of NS1 in the serum samples with an in-house DENV1 specific NS1 capture ELISA was performed according to the published protocol with minor modifications [8]. Briefly, microwell plates were coated with 100 μL/well of a MAb specific for DENV1 NS1 at a concentration of 10 μg/mL overnight at 4°C. After the blocking steps were performed, the 1:10 dilution of serum samples were added to duplicate wells (100 μL/well) and incubated for 1 h at 37°C. After the plates were washed, 100 μL/well of diluted HRP-conjugated MAb specific for DENV1 NS1 was added and incubated for 30 min at 37°C. After further washing, 100 μL/well of tetramethylbenzidine was added. The reaction was stopped after 10 min by the addition of 0.3 N sulfuric acid, and the plates were then examined in an ELISA plate reader. The cutoff value of the NS1 ELISA was determined by using 537 normal serum specimens from healthy donors. The cutoff value was set as the mean OD_450 _value of negative controls plus 5-times the SD. The result was considered positive if a sample yielded an OD_450 _value above the cutoff value.

### IgM and IgG detection with Panbio Dengue IgM and IgG capture ELISAs

Anti-dengue IgM and IgG antibodies were measured with the commercially available Panbio Dengue IgM capture ELISA (Cat. No. EDEN01M) and Dengue IgG capture ELISA (Cat. No. E-DEN02G). The results are classified as positive, negative and equivocal according to the manufacturer's instructions. The initial equivocal result was retested to confirm the result.

### Definition of primary or secondary infection with Panbio Dengue IgM and IgG capture ELISAs

The infection status was determined by Panbio Dengue IgM and IgG capture ELISA as described above according to the manufacturer's instructions. According to the published criteria, the infection status was classified as follows: a serum positive for IgM antibody and negative for IgG antibody or a negative IgG test in a serum sample collected at least five days after disease onset, followed by seroconversion in the convalescent serum sample is considered as primary infections; a positive or negative serum for IgM antibody, but positive for IgG antibody test for an acute-phase sample obtained within 4 days of disease onset is considered as secondary infections [15-17].

## Results and discussion

**Figure 1:**
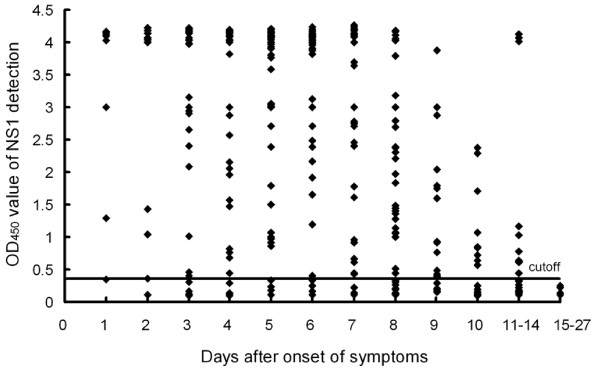
**Detection of NS1 in 313 serum specimens from 140 DENV1 infected patients**. Data represent the OD_450 _value of serum samples tested at 1:10 dilution. The solid line represents the cutoff value. The result was considered positive if a sample yielded an OD_450 _value above the cutoff.

**Figure 2:**
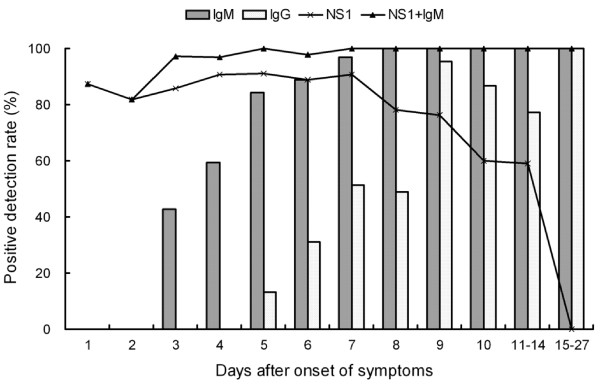
**Dynamics of dengue NS1, IgM and IgG antibody responses in DENV1 primary infection**.

In this study, we analyzed 313 paired or multiple serum samples from 140 patients with laboratory confirmation of acute DENV1 infection by using the DENV1 NS1 ELISA, dengue IgM and IgG ELISA. All of these patients were classified as the primary infection based on the interpretative criteria described above. As shown in Figure 1, high levels of NS1 in acute-phase samples from the primary infected patients were demonstrated. Dengue NS1 was detectable as early as the first day after the onset of illness with high positive rate of 87.5%. The overall sensitivity of detection was 89.0% (186 of 209) with samples taken during the first 7 days and 70.7% (70 of 99) for samples taken 8-14 days after the onset of symptoms. NS1 was not detectable beyond day 14 after the onset of disease (Figure 2). In contrast to NS1 detection, anti-dengue IgM and IgG antibodies were not detectable before day 3 of illness. IgM was detectable with the positive rate of 42.9% by the third day of illness, and rapidly increasing to 100% by day 8 of illness. The positive rate of IgG was significantly lower than that of IgM at the first week of onset, and slowly increasing to 100% by day 15 of illness. Combining the results of NS1 and IgM detection allowed positive diagnosis in 96.9% -100% for samples taken after day 3 of onset.

The present study is the first report of kinetics of NS1, simultaneously, IgM and IgG responses over the course of the disease in DENV1 primary infected patients. A primary dengue infection has been characterized by a slow and low titer of IgG antibody response. IgM antibodies appear only 3 to 5 days after onset of the disease [2-4,18]. Thus, there is a transient window period of first few days of illness if antibody is used as a diagnostic test. NS1 detection has shortened the window period by a positive result preceding and later overlapping positive detection of antibody. It is therefore no doubt that NS1 is a promising early diagnostic maker. The combination of NS1 and IgM testing facilitates enhanced diagnosis rates in acute- and early convalescent-phases of infection.

The levels of NS1 antigen might reflect the viral load during the course of disease as demonstrated by others [11]. The NS1 circulating in a patient's blood is longer periods than does viral RNA [7,12,19]. Therefore, detection of NS1 antigen may afford a valuable diagnostic test during the clinical phase where viral RNA is not detectable. A limitation of the present study was a lack of secondary infection samples. It has been demonstrated previously that the sensitivity of NS1 detection is significantly higher in acute primary dengue than in acute secondary dengue [11,20]. In secondary dengue infection, circulating NS1 antigen detection may be affected by the earlier elicited high titers of IgG antibodies.

## Conclusions

This study shows the kinetic profiles of circulating NS1, dengue IgM and IgG antibody responses as measured by ELISA-format assays of samples taken on different days after onset of symptoms. This work described here demonstrated that dengue NS1 antigen is a very promising early diagnostic marker. However, laboratory diagnosis must consider the timing of the clinical course, a strict diagnosis of acute dengue infections requires a combination of several tests performed at different stages of the disease.

## Competing interests

The authors declare that they have no competing interests.

## Authors' contributions

DH performed the NS1, IgM and IgG assays, analyzed the data and jointly drafted the manuscript. BD and MW collected serum samples and identified dengue infection by performed RT-PCR, virus isolation and serology. XD, YW, and KW jointly performed the NS1, IgM and IgG assays. YC and YP optimized the NS1 capture ELISA. XC conceived and designed the study, and drafted the manuscript. All authors read and approved the final manuscript.
